# The introduction of a breast cancer screening programme in a region with a population at medium risk for developing breast cancer: Khanty-Mansiysky autonomous Okrug—Ugra (Russian Federation)

**DOI:** 10.3332/ecancer.2011.195

**Published:** 2011-02-14

**Authors:** N Zakharova, SW Duffy, J Mackay, E Kotlyarov

**Affiliations:** 1 State Clinical Hospital, Khanty-Mansiysk, Khanty-Mansiysky Autonomous Okrug—Ugra, Russian Federation; 2 Cancer Research UK, Centre for Epidemiology, Mathematics and Statistics, Wolfson Institute of Preventive Medicine, Barts and The London School of Medicine and Dentistry, Queen Mary University of London, UK; 3 Research Department of Genetics, Evolution and Environment Faculty, University College London, UK; 4 Khanty-Mansiysk State Medical Academy, Khanty-Mansiysk, Khanty-Mansiysky Autonomous Okrug—Ugra, Russian Federation

## Abstract

**Background:**

A breast cancer screening programme (BCSP) was started in 21 districts in the Khanty-Mansiysky Autonomous Okrug—Ugra region from 7 February 2007.

**Methods:**

From the data on the numbers of subjects screened and the resulting diagnoses we calculated screening coverage rates and cancer detection rates and estimated the sensitivity of the screening tests. The State Cancer Registry was the source for the data regarding the female population by age, the number of breast cancer cases and cancer-related deaths. The data pertained to the period 2002–9, and included pathology data. Disease rates and distribution graphs of tumours by size and node status were analysed using Poisson regression.

**Results:**

The rates of breast cancer incidence and mortality indicate that the region is one in which the population is at medium risk of developing the disease. There was a significant increase in incidence overall during the period studied (*p* = 0.03), and a significant downward trend in breast-cancer-related mortality to a greater extent in the 50+ age group (*p* = 0.004), and when all ages 40+ were considered (*p* < 0.001). During 2007–9 92,576 women were screened in the BCSP. The screening coverage rate was ∼30%. Of the women who were screened, 9% were referred for further assessment. The average cancer detection rate was 2.5 per 1000 women screened. The sensitivity of the test was estimated to be 74%. Approximately 90% of screening-detected cases of breast cancer were at stages 1 or 2 of the disease.

**Conclusion:**

The finding that screening led to tumours being detected at earlier stages of the disease suggests that, in the long term, the programme will be successful in achieving a further reduction in mortality from the disease.

## Background

Breast cancer is a leading form of malignancy in the female population and is the main cause of death in women from the age of ∼40 years onwards in the USA [1]. The detection of breast cancer in the early stages is the main aim of screening programmes [2]. If diagnosed and treated at an early stage, breast cancer has a very high 5-year survival; also, early detection gives the opportunity for successful treatment using less aggressive methods.

The main goal of the randomized trials of breast cancer screening was to obtain a clear answer to the question: does mammography screening lead to lower rates of breast cancer-related mortality? Eight classic randomized trials of breast cancer screening were reviewed by Smith *et al* [3], and the report on a further trial has been published subsequently [4]. The trials provided conclusive evidence that the policy of offering screening is associated with a significant reduction in mortality on account of breast cancer. Among all invited (attended and non-attended) the breast cancer-related mortality was 20%, which is of high statistical significance. The screening trials invited subjects to participate, and 70% of those invited actually attended for screening. The mortality rate relating to breast cancer was estimated to be 30% lower among the women who underwent screening. In these screening trials, the recall rate on account of doubtful results of mammograms was acceptably low (5–6% at first screen and 2.5–3%) [5,6].

The trial results provide proof-of-principle of the efficacy of conducting screening programmes to detect breast cancer. When a screening service is set up in the routine health-care system rather than in the research setting, there is a need to evaluate its effect on breast cancer incidence, disease stage at the time of detection, and mortality. In this situation, it is important to have data on the incidence, stage-specific incidence and breast cancer-related mortality in the target population before the implementation of the screening programme. This information makes it possible to estimate changes brought about by the screening programme.

In Russia, the most extensive programme of screening for early detection of breast cancer is currently being conducted in Moscow [7]. This programme was started in a small number of hospitals in 1998, but full implementation throughout Moscow began in 2004. The programme offers screening at 2-year intervals to women 40–60 years of age. Screening is by 2-view mammography and the films are single-read. During the period 2004–7, 1.3 million women were screened and 3311 cases of breast cancer were detected (2.49 per 1000 screened). Because it was found that 43% of the women with breast cancer were 40–60 years of age while 25% were 60–69 years of age, it is planned to extend the age range for screening to 69 years.

Since 7 February 2007, a breast cancer screening programme (BCSP) is being implemented in 21 districts in the Khanty-Mansiysky Autonomous Okrug—Ugra (Ugra). This is an area in western Siberia covering 534,800 square kilometres, with a population of ∼1.5 millions.

## Methods

### Organization of the BSCP

**Figure 1:**
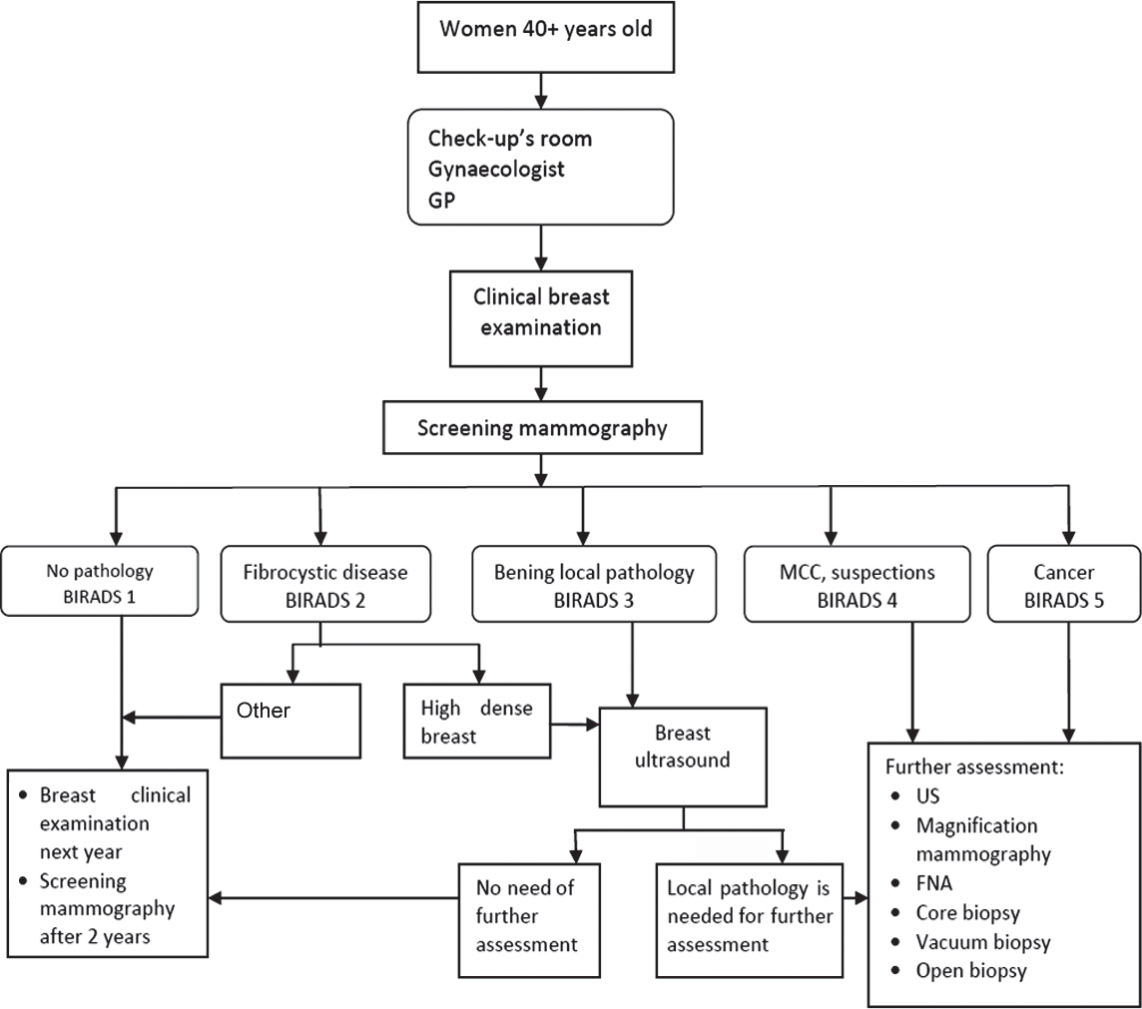
Organizational structure of the Breast Cancer Screening Programme in Ugra.

The BCSP has several consecutive steps (Figure 1). The first step takes place locally and may be in remote regions without local oncology facilities: here, gynaecologists or medical assistants perform clinical breast examination (CBE) and requisition mammography for all women over the age of 40 years. The second step involves instrumental diagnostics for workup of suspicious findings at the initial screening. The third step involves further diagnostic and treatment measures by oncologists in women in whom local breast pathology has been detected during the second step.

The BCSP covers women over the age of 40 years. Two-view mammography and single reading are the standard for screening, with 2-year intervals between successive screenings. The eligibility criteria are: all women 40 years and above in age, who have not undergone mammography in the previous 2 years and have not previously been diagnosed with breast cancer.

The stationary mammography units are located in 21 districts of Ugra in State clinical hospitals, local hospitals and outpatient departments. There are two mobile units as well as one unit on a boat that serves as a mobile outpatient department.

All the readers of the mammography screens are certified radiologists. The readers at the State clinical hospitals and at some of the regional hospitals have adequate previous experience with interpreting mammograms. The assistants in the mammography units at local hospitals and outpatient departments were trained at the State clinical hospitals before or during the first year of implementation of the BCSP. As part of standardization of the screening data BIRADS classification is now being used at the State clinical hospital in Khanty-Mansiysk.

In most of the hospitals in Ugra, the radiologists do not have the responsibility for carrying out further assessment after the mammogram. Women who need further medical assessment are referred to a medical oncologist. All newly detected breast cancer cases are discussed at the multidisciplinary team conferences in the regional oncology centres. The team includes a medical oncologist, a chemotherapist, a radiologist and an oncology surgeon. As a result of this system of holding conferences, each patient receives an individual treatment plan. Surgery and chemotherapy are offered in the three regional oncology centres in the Ugra, while radiotherapy is available at oncology centres outside the Ugra (Tyumen, Ekaterinburg).

For women who are eligible for medical insurance from the regional insurance company, the two-view mammograms of both breasts, as well as further medical assessment, are free of charge.

### Recruitment of women for screening programmes

In other screening programmes, the usual way to recruit women to the programme is by sending personal invitation letters with information about the screening and offering appointments for clinical examination. This method is not feasible in the Ugra for two main reasons. First, there is considerable mobility of households, and second, it is very difficult to plan appointments for women from remote rural areas because of problems with the transport system and weather conditions (especially low temperature in the winter).

Therefore the women are given the invitation during their annual health check-up (annual cervical smear and CBE) or during their visit to a gynaecologist or GP.

### Data and evaluation methods

We obtained data relating to the female population, by age, from 22 districts; we also obtained data from the State Cancer Registry on the number of breast cancer cases and related deaths from 2002 to 2009. In addition, the registry provided pathology data on tumour size, lymph node status and the stage of the tumour as per the Tumour Node Metastasis staging system. From these data we calculated the effect of screening-related detection on the stage of the disease at diagnosis. We calculated age-specific (40–49 years and 50+ years) incidence and mortality rates. In addition to mortality rates, we also estimated the percentages of breast cancer-related deaths in terms of the time elapsed after diagnosis. This was because early deaths are usually attributable to the disease being at an advanced stage at the time of diagnosis, whereas later deaths may involve factors relating to the treatment. Analyses of disease rates and distribution graphs of tumours by size and node status were carried out using Poisson regression [8].

From the paper returns of the 21 districts, we have data on the numbers of women screened. Unfortunately, from these paper reports, we do not have data on screening activity specific to age or to the screening round (prevalent/incident). However, because the data pertain to the first 2 years of the programme, the overwhelming majority of the screening episodes would relate to prevalence screening. From this data, we report screening coverage rates. We also obtained data on the mode of detection of the breast cancers that were diagnosed (whether through screening or through report of symptoms), and estimated the effect of screening on the pathologies of the tumours diagnosed. We calculated screening-related detection rates, and from these we estimated the sensitivity of the screening test [9].

In addition to reporting the screening coverage rates, we also report diagnostic mammography coverage, as in the study by Boncz *et al* [10], using the following definitions:

$$\text{Screening}\;\text{coverage}\;(\%)=\frac{\text{Number}\;\text{of}\;\text{women}\;40+\text{years}\;\text{old}\;\text{having}\;\text{screening}\;\text{mammography}}{\text{Female}\;\text{population}\;40+\text{years}\;\text{old}}\times 100$$

$$\text{Diagnostic}\;\text{coverage}\;(\%)=\frac{\text{Number}\;\text{of}\;\text{women}\;40+\text{years}\;\text{old}\;\text{having}\;\text{diagnostic}\;\text{mammography}}{\text{Female}\;\text{population}\;40+\text{years}\;\text{old}}\times 100$$

$$\text{Total}\;\text{coverage}\;(\%)=\frac{\text{Number}\;\text{of}\;\text{women}\;40+\text{years}\;\text{old}\;\text{having}\;\text{either}\;\text{screening}\;\text{or}\;\text{diagnostic}\;\text{mammography}}{\text{Female}\;\text{population}\;40+\text{years}\;\text{old}}\times 100$$

## Results

It should be noted that the demographic profile of the female population in Khanty-Mansiysky Autonomous Okrug has changed dramatically during the period 2002–9. In 2002, of the 40+-age female population the majority was in the age group 40–49 years. In 2009, the majority of the women were >50 years of age (Table 1).

Table 2 gives the incidence and mortality rates pertaining to breast cancer in women ≥40 years of age in the Ugra, between 2002 and 2009. This shows that the region is one where the female population is at medium risk for developing breast cancer, with rates lower than those in Western Europe and North America but higher than those in East Asia. There was a significant increase in the incidence of breast cancer overall during the period (*p* = 0.03), although the trend within each age group was not significant. The increase in incidence rates occurred gradually in the 40–49 age group; however, in the 50+ group there were substantial increases in incidence in the years 2005 and 2006 (before the introduction of screening), followed by a fall in incidence thereafter. Overall, in women aged 40+ years there was no evidence of an increase in incidence of the disease associated with the introduction of the screening programme. Over the period of observation, there was a significant trend of falling breast-cancer-related mortality in the 50+ age group (*p* = 0.004), as well as overall (*p* < 0.001). However, there was a higher rate of mortality, against the trend, in the year 2008.

Table 3 shows the total number of mammography screenings (prevalence and incidence) by year. At this early stage in the programme, the majority of screenings will be prevalence screenings. As noted earlier, given that these figures are from paper returns from the 21 districts, we were unable to separate prevalence screenings from incidence screenings. The table also includes the numbers of total (diagnostic + screening) mammography examinations carried out. The calculation shows particularly high rates of diagnoses of breast cancer made on the basis of mammography findings.

The diagnoses of cancer resulting from screening are also shown in Table 3. From these, we can derive an estimate of the screening test sensitivity [9]. The screenings are mainly prevalence ones. The expected rate of detection of cancers at prevalence screening depends on the underlying incidence, the sensitivity of the test, and the mean sojourn time (the duration of the preclinical screen-detectable period). Our prevalence screening detected 2.5 cases of cancer per thousand women screened. As underlying incidence, we assume 1.23 per thousand, which was the incidence rate in the year immediately preceding the introduction of the programme. According to Paci and Duffy [11], the MST is estimated to be ∼1.5 years for women 40–49 years of age, and 4.0 years in women 50–69 years of age. We take the average of these, namely, 2.75 years as applicable to women in the 40+ age group in the study population. The sensitivity S can then be calculated as:

$$S=\frac{0.0025}{0.00123\times 2.75}=0.74$$

That is, we estimate the test sensitivity to be 74%.

**Figure 2:**
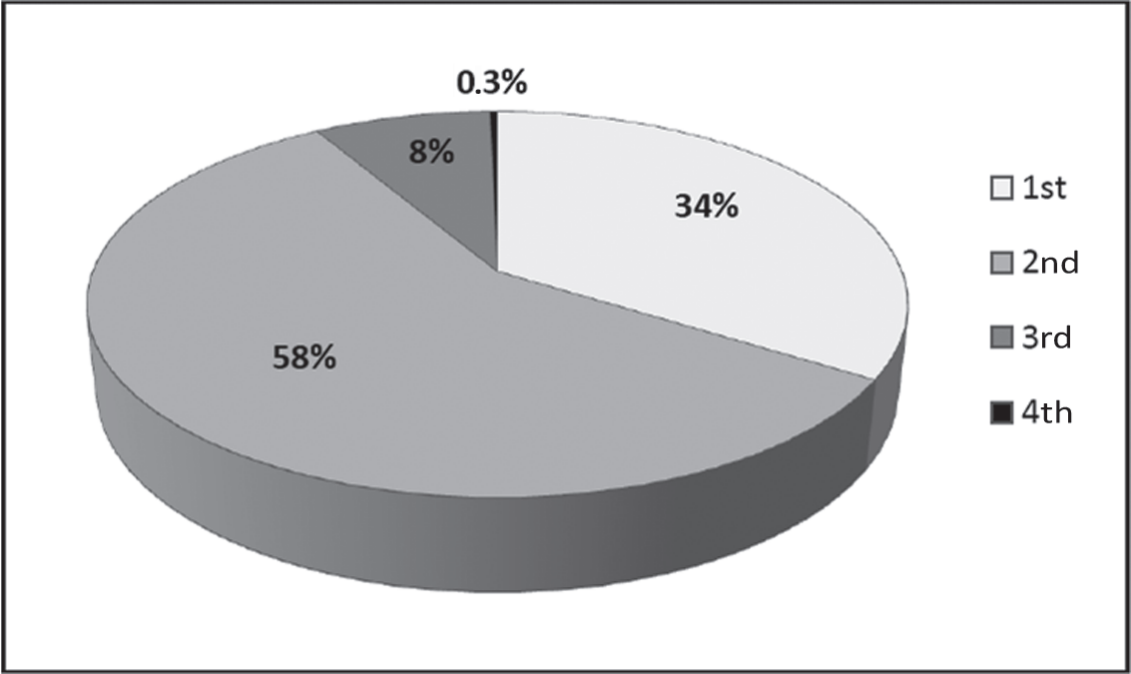
Screening-detected breast cancer cases, distributed by stage of the disease, 2007–9.

**Figure 3:**
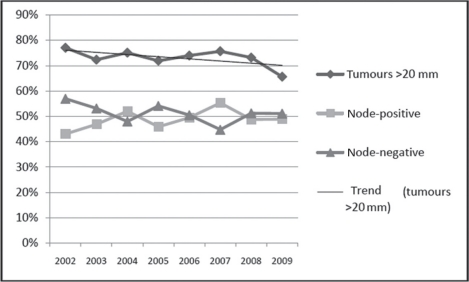
Size of tumour and node status of breast cancers in the Ugra screening programme in women in the age group 40+, detected in 2002–9.

Table 4 shows the pathology data relating to screening-detected and symptomatic cancers, by age group and year of diagnosis, from the State Cancer Registry. As expected, the screening-detected tumours are less likely to be larger than 20 mm and less likely to be node-positive. The differences in tumour size were significant, both in the 40–49 age group (*p* = 0.002) and in the 50+ age group (*p* < 0.001). As regards node status, the difference was not significant in the 40–49 age group (*p* = 0.2), but was significant in the 50+ group (*p* < 0.001). Table 4 shows that, in the 40–49 age group, the percentages of large and node-positive tumours among the symptomatic cases was relatively stable over time. It is too early to discern a clear trend among the screening-detected cases. However, ∼90% of all the cases detected through screening during 2007–9 were at stages 1 or 2 of the disease (Figure 2). The overall (screening-detected + symptomatic) percentage of tumours with size >20 mm decreased during the period 2002–9 (Figure 3).

**Figure 4:**
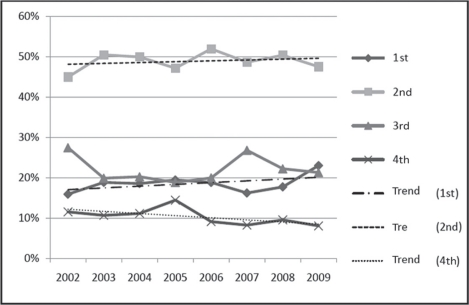
Stage-specific changes in breast cancer detection rates in women in the 40+ age group, 2002–9.

Table 5 shows breast cancer cases (symptomatic-detected + screening-detected) by calendar year (2002–9), and stages of the disease at the time of detection, for the two age groups. In both the age groups, there was a nonsignificant increase with time in the percentage of stage 1 and stage 2 tumours detected, and a decrease in stage 4 tumours detected (Figure 4). In the 40–49 age group, there was a statistically significant decrease in the percentage of tumours detected at stage 4 of the disease (*p* = 0.04).

Table 6 shows data for each of the age groups on deaths related to breast cancer, both by year of occurrence and by time elapsed since the diagnosis. There were no significant time-related trends. Around one-third of the deaths occurred within 2 years of diagnosis. In the year 2008, when there was a particularly high mortality rate in the 50+ group, a relatively low proportion of the breast cancer deaths were from tumours diagnosed within the previous 2 years, and a relatively high proportion were from tumours diagnosed 6 or more years earlier. The opposite phenomenon was observed in the 40–49 age group, wherein almost half of the deaths in 2008 were from tumours diagnosed within the previous 2 years.

## Conclusion

Results for screening programmes worldwide suggest that they are achieving substantial reduction in breast cancer-related mortality [12]. It is too early to estimate the effect on mortality in our programme; in this paper we have investigated the early indicators of the programme’s quality.

During 2007– 2009, in our BCSP, 92,576 women were screened. The screening coverage rate was ∼30% of the population of Khanty-Mansiysky Autonomous Okrug – Ugra region. The diagnostic mammography coverage rate was 18%. This is a high percentage, and possibly reflects a rise in awareness about breast cancer among the female population.

Approximately 9% of the women screened were recommended for further assessment. This number is within the range observed in Europe (varying between 1.3 and 18.4%) [13,14]. The average cancer detection rate was 2.5 per 1000 women screened, which was more than double the underlying incidence rate. The sensitivity of the screening mammography test was estimated to be 74%. This too is in line with sensitivity values observed in other screening programmes, particularly those that included women below the age of 50 years [14,15].

The BCSP was introduced against a background of medium rate of incidence of the disease, with some indication of a long-term trend towards increasing incidence. Interestingly, there was no substantial increase in incidence observed during the years 2007–9, thereby suggesting that overdiagnosis was not a problem in this programme. However, this aspect should be monitored in the future as coverage improves. In Italy, after 15 years of observation, very little overdiagnosis was seen [16]. There was evidence of a long-term trend towards improvement in detection and diagnosis at earlier stages of the disease, and towards a decrease in the breast cancer-related mortality rate. It is too early to estimate the health impact of the screening programme, but the fact that screening was able to detect tumours at earlier stages of the disease suggests that, in the long term, the programme will be successful in achieving further reduction in mortality from the disease.

The main problem for our programme is the low coverage rate. Therefore the first task is to improve enrolment in the programme among the target population in our region. The lack of information on the round of screening is not a problem for this evaluation because, up to now, almost all screenings will have been related to prevalence. In future, however, this shortcoming threatens to impair both the organization of the programme and the calculation of coverage and detection rates, both of which are highly important for the evaluation of the screening programme. Thus, the next task is to organize a comprehensive screening data capture system, to collect more detailed data on the screening process, including the round of screening, and information on interval cancers. Since the beginning of 2010 this information is required to be included in the report form to be sent to the State Cancer Registry about each newly detected case of cancer, and also in the annual report from x-ray departments to the Department of Healthcare.

The BCSP in Khanty-Mansiysky Autonomous Okrug—Ugra is the first experience in Russia of implementing a breast cancer screening programme in this large northern territory with a low population density (2.7 per sq. km) and medium risk for developing the disease. Screening sensitivity is adequate, and there is no indication of overdiagnosis to date. The cases detected through screening show more favourable characteristics as regards tumour size and node status, thereby suggesting that the desired mortality reduction will follow in the coming years. There is a need to improve coverage and to collect more data for evaluating the performance of the programme.

## Figures and Tables

**Table 1:** Female population age 40 years or more in the Ugra, 2002–9

**Age**	**Year**							
	**2002**	**2003**	**2004**	**2005**	**2006**	**2007**	**2008**	**2009**
40–49	146,184	147,290	149,384	152,901	147,300	145,365	140,787	142,963
50+	126,916	131,467	135,798	135,953	147,800	159,587	168,859	178,652
Total	273,100	278,757	285,182	288,854	295,100	304,952	309,646	321,615

**Table 2:** Breast cancer incidence and mortality by age group in Ugra in 2002–9 (per 100,000 female population)

**Year**	**Incidence**			**Mortality**		
	**40–49 years old**	**50+ years old**	**40+**	**40–49 years old**	**50+years old**	**40+**
2002	74.6	119.0	95.2	21.2	67.8	42.8
2003	81.5	129.3	104.0	24.4	70.7	46.3
2004	89.0	128.9	108.0	24.1	69.2	45.6
2005	85.7	162.6	121.9	20.3	67.7	42.6
2006	89.6	156.3	123.0	24.4	56.2	40.3
2007	93.6	132.8	114.1	16.5	55.1	36.7
2008	89.5	137.4	115.6	20.6	63.3	43.9
2009	79.7	134.9	110.4	18.2	38.1	29.2

**Table 3:** Screening activity and diagnoses as a result of screening, 2007–9

	**2007**	**2008**	**2009**	**Total**
Number of all mammography examinations (diagnostic + screening)	38,902	56,757	53,347	149,006
Number of screening mammography examinations	19,982	35,243	37,351	92,576
Local pathology (fibroadenoma or adenosis, or other findings)	2291	2315	3766	8372
Breast cancer (rate per 1000 screened)	36 (1.8)	107 (3.0)	84 (2.2)	227 (2.5)

**Table 4:** Size and node status of symptomatic and screen-detected tumours

**Years**	**Total number**		**Tumours >20 mm (%)**		**Node-positive (%)**	
	**40–49 years old**	**50+ years old**	**40–49 years old**	**50+ years old**	**40–49 years old**	**50+ years old**
2002–6 Symptomatic-detected	547	766	383 (70)	588 (77)	244 (45)	383 (50)
2007–9 Symptomatic-detected	278	455	201 (72)	354 (78)	149 (54)	254 (56)
2007–9 Screen-detected	76	140	41 (54)	82 (58)	29 (38)	51 (36)
2007–9 Total detected	354	595	242 (68)	436 (73)	178 (50)	305 (51)

**Table 5:** Breast cancers distributed by stage in the Ugra, 2002–9

**Year**	**Stage 1 (%)**		**Stage 2 (%)**		**Stage 3 (%)**		**Stage 4 (%)**	
	**40–49 years old**	**50+ years old**	**40–49 years old**	**50+ years old**	**40–49 years old**	**50+ years old**	**40–49 years old**	**50+years old**
2002	19 (18)	26 (15)	49 (46)	69 (44)	29 (27)	34 (27)	9 (9)	10 (14)
2003	27 (23)	23 (16)	51 (43)	52 (56)	24 (20)	41 (20)	17 (14)	13 (8)
2004	26 (20)	29 (17)	72 (56)	76 (46)	21 (16)	39 (23)	10 (8)	23 (14)
2005	31 (25)	35 (16)	57 (45)	103 (49)	25 (20)	39 (18)	13 (10)	36 (17)
2006	29 (22)	37 (17)	72 (55)	110 (50)	23 (18)	47 (21)	6 (5)	26 (12)
2007	30 (23)	25 (12)	58 (44)	107 (52)	38 (29)	53 (26)	6 (4)	22 (10)
2008	20 (16)	43 (19)	63 (50)	116 (51)	31 (25)	48 (21)	12 (9)	22 (9)
2009	29 (26)	51 (22)	59 (53)	106 (45)	19 (17)	55 (23)	5 (4)	23 (10)

**Table 6:** Breast cancer deaths by year, age and time since diagnosis

	**1–2 years since diagnosis No. (%)**			**3–5 years since diagnosis No. (%)**			**6+ years since diagnosis No. (%)**		
	**40–49 years old**	**50+years old**	**40+years old**	**40–49 years old**	**50+years old**	**40+years old**	**40–49 years old**	**50+years old**	**40+years old**
2002	13 (42)	26 (30)	39 (33)	10 (32)	28 (33)	38 (32)	8 (26)	32 (37)	40 (35)
2003	15 (42)	29 (31)	44 (34)	13 (36)	38 (41)	51 (40)	8 (22)	26 (28)	34 (26)
2004	13 (36)	26 (28)	39 (30)	12 (33)	35 (37)	47 (36)	11 (31)	33 (35)	44 (34)
2005	8 (26)	37 (40)	45 (37)	13 (42)	25 (27)	38 (31)	10 (32)	30 (33)	40 (32)
2006	14 (39)	30 (36)	44 (37)	17 (47)	33 (40)	50 (42)	5 (14)	20 (24)	25 (21)
2007	8 (33)	22 (25)	30 (26)	8 (33)	33 (38)	41 (37)	8 (33)	33 (37)	41 (37)
2008	14 (48)	28 (26)	42 (31)	8 (28)	40 (37)	48 (35)	7 (24)	40 (37)	47 (34)
2009	10 (39)	18 (27)	28 (30)	12 (46)	28 (41)	40 (42)	4 (15)	22 (32)	26 (28)
